# A High-Performance All-Carbon Diamond Pixel Solar-Blind Detector with In Situ Converted Graphene Electrodes

**DOI:** 10.3390/ma18061222

**Published:** 2025-03-10

**Authors:** Mingxin Jiang, Zhenglin Jia, Mengting Qiu, Xingqiao Chen, Jiayi Cai, Mingyang Yang, Yi Shen, Chaoping Liu, Kuan W. A. Chee, Nan Jiang, Kazuhito Nishimura, Qingning Li, Qilong Yuan, He Li

**Affiliations:** 1 School of Material Science and Engineering, Guilin University of Electronic Technology, Guilin 541004, China; jiangmingxin@nimte.ac.cn; 2 State Key Laboratory of Advanced Marine Materials, Ningbo Institute of Materials Technology and Engineering, Chinese Academy of Sciences, Ningbo 315201, China; jiazhenglin@nimte.ac.cn (Z.J.); chenxingqiao@nimte.ac.cn (X.C.); caijiayi@nimte.ac.cn (J.C.); yangmingyang@nimte.ac.cn (M.Y.); shenyi@nimte.ac.cn (Y.S.); liuchaoping@nimte.ac.cn (C.L.); jiangnan@nimte.ac.cn (N.J.); kazuhitonishimura@nimte.ac.cn (K.N.); 3 Southwest Institute of Technical Physics, Chengdu 610041, China; 18183312062@163.com; 4 National Laboratory for Physical Sciences at Microscale, University of Science and Technology of China, Hefei 230026, China; kuan.chee@cantab.net; 5 Center of Materials Science and Optoelectronics Engineering, University of Chinese Academy of Sciences, Beijing 100049, China

**Keywords:** all-carbon detector, single-crystal diamond, graphene electrodes, pixel array, solar-blind imaging

## Abstract

Solar-blind ultraviolet detectors, known for their low background noise and high sensitivity, have garnered significant attention in various applications such as space communications, ozone layer monitoring, guidance applications, and flame detection. Pixel photodetectors, as the cornerstone of imaging technology in this field, have become a focal point of research in recent years. In this work, a solar-blind photodetector with a 6 × 6 planar pixel array was fabricated on single-crystal diamond substrate, utilizing in situ conversed graphene electrodes. The graphene electrodes achieved exceptional Ohmic contact with the diamond surface, boasting a remarkably low specific contact resistance of 6.73 × 10^−5^ Ω·cm^2^. The diamond pixel detector exhibited high performance consistency with an ultra-low dark current ranging from 10^−11^ to 10^−12^ A and a photocurrent of 10^−8^~10^−9^ A under 222 nm illumination with a bias of 10 V. This work not only demonstrates the feasibility of fabricating all-carbon solar-blind photodetectors on diamond but also highlights their potential for achieving high spatial resolution in solar-blind image detection.

## 1. Introduction

Solar-blind ultraviolet (UV) detectors, used for detecting UV light with wavelengths smaller than 280 nm, have broad application in both national defense and civil fields [1,2,3], as UV light smaller than 280 nm in sunlight cannot reach the earth surface due to the adsorption of the ozone layer. Wide and ultrawide bandgap semiconductors, including SiC [1], AlN [2,3], Al_X_Ga_1−X_N [4,5], Mg_X_Zn_1−X_O [6,7], Ga_2_O_3_ [8,9], and diamond [10,11,12,13,14,15], have been utilized to fabricate solar-blind detectors due to their wide bandgap, which can suppress the response to visible light and achieve small size, light weight, and strong anti-interference ability [16,17,18]. The newly discovered Tutton salts are also expected to be developed [19,20]. Among these materials, diamond is regarded as one of the most promising materials, as its bandgap is high, up to 5.47 eV, ensuring the ultra-low intrinsic carrier concentration (intrinsic diamond resistivity up to 10^6^ Ω·cm^2^ at room temperature) [21] and ultral-low dark current when fabricated into photodetectors. Numerous diamond-based photodetectors have been fabricated for solar-blind UV detectors [12,13,14]; however, nowadays, few diamond detectors can used for solar-blind imaging [22].

Due to the above excellent properties of diamond, photodetectors with diamond-based materials have been developed rapidly in recent decades. For example, Zhang prepared ultraviolet (UV) photodetectors by depositing single-walled carbon nanotubes (SWCNTs) on the surface of graphene field-effect transistors (GFETs) with a buried gate electrode structure, which had an extremely high photoresponsivity of up to 204.5 A/W under the irradiation of an LED at 365 nm with an incident power of 3.9 µW [23]. Li prepared a photodetector with a laser-induced in situ structural transformation from sp^3^ heterozygous carbon to sp^2^ heterozygous carbon. Li converted the in situ structure of sp^3^-hybridized carbon into sp^2^-hybridized carbon-prepared all-carbon electrodes, and the all-carbon photodetectors had a high responsivity of 15 mA/W and a fast transient response of 86 μs under 220 nm light illumination [24]. Zhang prepared a detector array with 6 × 6 cells using polycrystalline diamond. This photodetector array had a response rate of 255 mA/W at 218 nm wavelength and 30 V bias with a relatively fast response (1.2 ms/0.51 ms). Clear images can be obtained utilizing the planar photodetector array as the sensor unit of the imaging system [25].

In this work, an all-carbon diamond photodetector with 6 × 6 planar pixel arrays with graphene as electrodes was fabricated for solar-blind imaging. Graphene electrodes were synthesized on diamond directly through in situ sp^3^-to-sp^2^ conversion with the assistance of nickel catalyst. The results showed that the graphene electrodes achieved excellent Ohmic contact on diamond surface with a specific contact resistance as low as 6.73 × 10^−5^ Ω·cm^2^. The photocurrent/dark current switching ratio and the peak responsivity of the all-carbon diamond detector were 10^2^ and 1.35 × 10^−4^ A/W with a bias voltage of 10 V, respectively. The all-carbon diamond pixel detector can realize the high-fidelity imaging display of deep ultraviolet images. This study provides an effective method for fabricating diamond photodetectors for UV imaging.

## 2. Experiment

A single-crystalline diamond with a size of 8 × 8 mm^2^ was epitaxially grown on homogeneous substrate in a home-made microwave plasma chemical vapor deposition system (MPCVD). A mixture of H_2_ (400 sccm) and CH_4_ (12 sccm) gases with high purity was pumped into the MPCVD system as growth precursor. After 10 h of growth at 930 °C, the single-crystal diamond was immersed in concentrated sulfuric acid and nitric acid solution (1:1 by volume) for 2 h to remove the contaminants on the diamond surface, followed by immersion in concentrated sulfuric acid and hydrogen peroxide solution (7:3 by volume) for 2 h to oxidize the surface sufficiently. Finally, it was ultrasonically cleaned with acetone, anhydrous ethanol, and deionized water in sequence.

After lithography and the development of photoresist on the diamond, Ni film with thickness of 15 nm was deposited on the diamond with an electron-beam deposition system (DZS500, SKY Technology Development Co., Ltd., Shenyang, China). After the removal of the photoresist, the diamond sample was rapidly annealed at 1000 °C in an argon atmosphere for 15 min for the formation of graphene electrodes on the diamond surface.

The crystallinity and transmittance of the prepared CVD diamonds were characterized using a confocal laser microscopic Raman spectrometer with a laser at a wavelength of 532 nm (LabRAMHR Evolution, Horiba, Japan) and a UV-visible spectrophotometer (Lambda 950, PerkinElmer, CT, USA), respectively. The surface morphology of the diamond samples was characterized with an atomic force microscope (AFM, Bruker Dimension ICON SPM, USA) and a confocal laser scanning microscope (Japan). A Xenon lamp (EQ-99X LDLSTM, Energetiq, USA) connected to a monochromator (iHR 320, Horiba, USA) was used to generate UV light with different wavelengths. The *I*-*V* curves of the diamond detector with and without UV exposure were measured with a Keithley 4200A-SCS (Tektronix, Shanghai, China).

## 3. Results and Discussion

### 3.1. Diamond Characterizations

**Figure 1:**
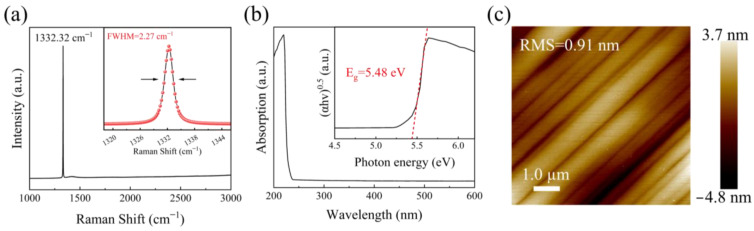
(**a**) Raman spectrum, (**b**) absorption spectra, and (**c**) AFM images of single-crystal diamond. The inset in (**a**) is the enlarged Raman peaks of the diamond. The insert in (**b**) is the tauc plot of the diamond.

The crystallinity and surface morphology of the diamond sample after CVD growth are characterized in Figure 1a. On the Raman spectroscopy, the sharp peak located at 1332.32 cm^−1^ is the phonon scattering peak of diamond crystal. The maximum half-peak width (FWHM) of the diamond peak is about 2.27 cm^−1^, indicating the good crystallinity of the CVD diamond. Besides the diamond typical peak, the small peak at around 1420 cm^−1^ can be attributed to nitrogen vacancy centers from the substrate. The absorption spectrum of the CVD diamond is shown in Figure 1b, in which the sharp absorption edge occurs at around 220 nm. The cutoff wavelength for visible transparency can indicate the selectivity of the optical response in practical applications. The optical energy bandgap of CVD diamond can be derived from the cutoff wavelength of the adsorption spectrum according to the Tauc plot from the following formula:

(1) $${\left(\alpha \mathrm{hv}\right)}^{n}=A\left(\mathrm{hv}-E\right)$$

where *α*, *h*, *v*, *A*, and *E* represent the absorption coefficient, Planck’s constant, incident light frequency, semiconductor forbidden bandwidth, and material-dependent constants, respectively. The exponent *n* is 0.5 for indirect bandgap semiconductors, including diamond [26]. As shown the tauc plot in the inset of Figure 1b, a bandgap of 5.48 eV can be derived, which is close to the theoretical value of 5.47 eV. The surface morphology of the grown CVD diamond was characterized by AFM after careful polishing, as shown in Figure 1c, in which the surface roughness of the diamond is about 0.91 nm.

### 3.2. Formation of Graphene Electrodes

**Figure 2:**
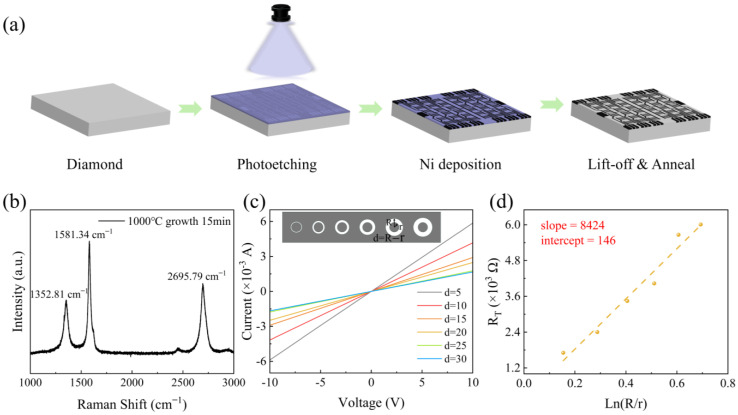
(**a**) Fabrication process of diamond solar-blind pixel photodetector with graphene electrodes, (**b**) Raman spectrum of graphene layer on diamond, (**c**) *I*-*V* curves of the cyclic transmission line model (CTLM), and (**d**) *R*_T_ and *Ln*(*R*/*r*) properties of graphene on diamond. The insets in (**c**) show the optical images of the circular transmission line model (CTLM).

**Figure 3:**
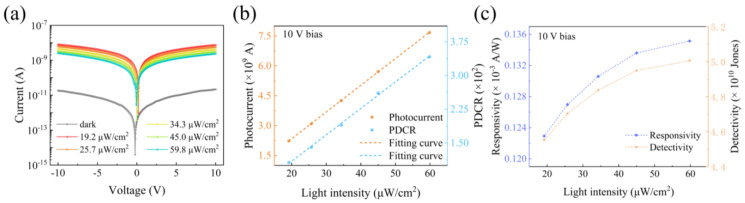
(**a**) Semi-logarithmic current–voltage curves of the device under dark conditions and illuminated with different 222 nm power intensities, (**b**) photocurrent and *PDCR* curves versus incident light intensity, and (**c**) dependences of *R* and D* on light intensity.

The graphene electrodes were fabricated by in situ annealing nickel deposited diamond at 1000 °C for 15 min [27], as shown in Figure 2a. An enlarged image of a single photodetector unit is shown in Figure S1, which consists of four pairs of interdigital electrodes with a width of 10 µm, a length of 180 µm, and a spacing of 20 µm. The effective area of each detector pixel and the distance between two neighboring photodetector units are 0.034 mm^2^ and 300 µm, respectively [28,29]. Clear and complete edge stripping in lithographically deposited nickel metal was ensured. The Raman profile of the in situ sp^3^-to-sp^2^-conversed graphene layers was characterized, as shown in Figure 2b, in which the characteristic G and 2D bands of graphene could be clearly observed at 1580 cm^−1^ and 2700 cm^−1^, respectively. The peak at 1350 cm^−1^ is the D band of graphene, which is associated with the defects in the graphene layers. To verify the electric contact properties of the graphene electrode on diamond, a circular transmission line model (CTLM) was constructed, as shown in the inset of Figure 2c. The current–voltage (*I-V*) characteristics were measured using excimer lamps with light conditions of 222 nm, as shown in Figure 3c. The specific contact resistance of graphene electrodes on diamond can be calculated with the following formula:

(2) $$R_{T}=\frac{R_{S}}{2\pi }\left[Ln\left(\frac{R}{r}\right)+L_{T}\left(\frac{1}{R}+\frac{1}{r}\right)\right]$$

where *R*_T_, *R*_S_, and *L*_T_ are the square resistance, measured resistance, and transmission line length, respectively [30]. In fitting *R*_T_ and *Ln*(*R*/*r*) linearly, as shown in Figure 2d, the specific contact resistance *R*_S_ × *L*_T_^2^ can be calculated from the intercept and slope. In this work, the specific contact resistance of graphene on the diamond surface was calculated to be about 6.73 × 10^−5^ Ω·cm^2^, which is similar to the contact properties of metals on the diamond surface, like Ti/Au [31,32] and Cr/Au [33,34].

### 3.3. Performance Characterization of Photodetectors

The incident UV power intensity of the diamond detector was tested, as shown in Figure 3; with the incident UV power intensity varying from 19.2 to 59.8 µW/cm^2^, the photoresponses of the diamond detector achieved symmetric curves at the negative and positive bias voltage, as shown in Figure 3a. The dependence of the photocurrent versus incident light power intensity was extracted and replotted in Figure 3b (orange curve). It can be seen that the photocurrent shows a nearly linear relationship to the incident UV power intensity, indicating that the photocurrent response increases with the increase in incident UV power intensity [35]. The light/dark current ratio (*PDCR*) is another important factor that is used to judge the photodetector’s noise immunity. It was determined by

(3) $$\mathrm{PDCR}=\frac{I_{\mathrm{photo}}-I_{\mathrm{dark}}}{I_{\mathrm{dark}}}$$

where *I*_photo_ and *I*_dark_ are the photo current and dark current of the diamond detector. In this work, the *PDCR* of the diamond detector showed an increasing tendency with the increase in the incident UV power intensity, as shown in the blue curve in Figure 3b, in which the *PDCR* increased from 106 to 341 when the incident UV power intensity increased from 19.2 µW/cm^2^ to about 59.8 µW/cm^2^.

In order to further quantitatively evaluate the optoelectronic performance of the fabricated diamond detectors, the responsivity (*R*), detectivity (D*), and rejection ratio of the detectors were measured and calculated [36]. The general responsivity *R*, also called sensitivity, is defined as

(4) $$R=\frac{I_{photo}-I_{dark}}{P_{in}}$$

where *P*_in_ is the incident optical power of the diamond detector. The measured maximum responsivity was 1.35 × 10^−4^ A/W at 59.8 µW/cm^2^, which is similar to that of other diamond detectors using Ti/Au electrodes.

In addition, D* is another important parameter used to evaluate the detection limit ability of the photodetector, which is related to the background noise, including shooting noise, flicker noise, etc. D* is determined by responsivity *R*, dark current *I*_dark_, and effective area *S* of the detector:

(5) $$D^{*}=\frac{R}{\sqrt{2qI_{dark}/S}}$$

When the bias voltage was 10 V and *P*_in_ = 59.8 µW/cm^2^, the calculated D* was 5.0 × 10^10^ Jones. The dependences of the *R* and D* to the incident UV light power intensity were calculated and plotted in Figure 3c, in which both *R* and D* increases tended to reach an equilibrium state with the increased light intensity. The low responsivity and detectivity of the fabricated all-carbon diamond detector can be related to the poor quality of the diamond, which contains many defects or impurities in bulk. According to the trend plots at different light intensities, both *R* and D* increase sharply with increasing light intensity. However, at higher light intensities, they increase slowly and converge to an equilibrium state. Since single-crystal diamond cannot avoid dislocation defects during growth, these defects trap charge carriers. As the photogenerated carriers are generated and recombined at higher light intensities, the photogenerated carrier generation and recombination will gradually reach a dynamic equilibrium, and the responsivity and specific detection rate of the diamond will increase. The response rate and specific detection rate of the device tend toward equilibrium.

**Figure 4:**
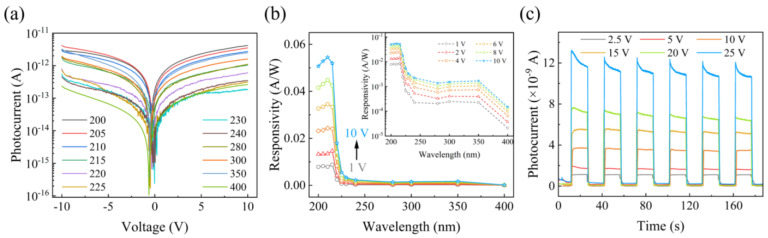
(**a**) Semi-logarithmic current–voltage curves of the device under 200~400 nm optical excitation, (**b**) spectral response plots at different bias voltages, and (**c**) time–photocurrent variations in diamond solar-blind pixel photodetectors at different biases.

The spectra response of the diamond detector was tested, as shown in Figure 4. The different UV wavelengths varying from 200 to 400 nm were generated from a continuous monochromatic source. The *I*-*V* curves of the diamond detector at different wavelengths are shown in Figure 4a. The responsivity of the typical diamond detector firstly increased and then decreased with the incident UV wavelengths increasing from 200 nm to 400 nm. The maximum responsivity of the diamond detector to UV light appeared at around 210 nm, and it decreased to almost zero when the UV wavelength increased over 280 nm. Meanwhile, according to the spectral response Figure 4b, it can be seen that the bias voltage increases from 1 to 10 V, and the device’s responsivity also increases gradually, which is attributed to the increase in the carrier drift field strength that leads to the improvement of the collection efficiency of photogenerated carriers. The inset in Figure 4b shows the semi-logarithmic spectral response at different bias voltages, where the optimum induced light intensity can be observed [22,37]. The solar-blind (*R*_210_/*R*_280_) suppression ratio of the diamond detector was calculated to be about 40, according to the insert in Figure 4b. The time-dependent response of the diamond detector was performed for six cycles under 222 nm of UV light illumination at different bias voltages. As shown in Figure 4c, the device exhibits reversible modulation under periodic UV exposure, demonstrating the reproducibility and stability of the diamond detector. The results show that the device meets the response in the UV region (200~280 nm) and has good repeatability and stability.

### 3.4. Solar-Blind Imaging Performance of Photodetectors

**Figure 5:**
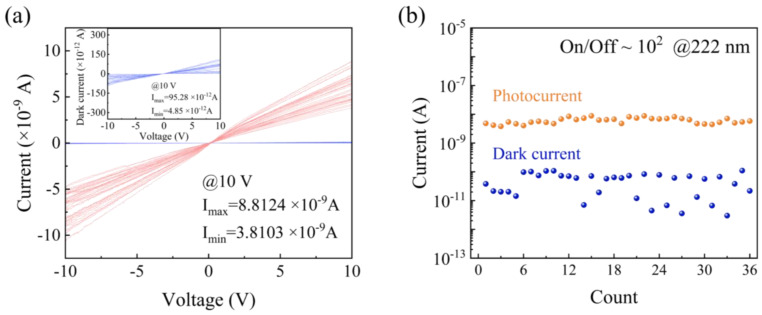
(**a**) The dark current and the photocurrent of the current–voltage curves of 36 detector units; the illumination wavelength and power were 222 nm and 59.8 µW/cm^2^, respectively. (**b**) The light/dark current ratio of the 36 detector units.

The photoresponse of the diamond detector was measured with and without UV illumination, as shown in Figure 5. The dark current and photocurrent of a typical diamond detector unit was measured, as shown in Figure 5a; both currents show a good linear relationship with the applied bias voltage, which also confirms the well Ohmic contact of graphene electrodes on the diamond surface. The current–voltage (*I*-*V*) curves of the 36 detector units were then measured under dark and 222 nm UV illumination, As shown in Figure 5a. At bias voltage of 10 V, the photocurrent of the 36-pixel arrays varied between 3.81 and 8.81 nA, and the dark current fluctuated within the range of 4.85~95.28 pA (the insert in Figure 5a), indicating that all photodetectors are functional and capable of being used as pixels in a planar array of photodetectors. Figure 5b shows the light/dark current ratio of the devices. The light/dark current ratios of the 36-pixel arrays on diamond were replotted, as shown in Figure 5b, where the 36 detector units show good uniformity in photocurrent compared to the dark current. The average *PDCR* of the 36 units was about 100. These results demonstrate the suitability of the device and the fact that the dark current measurements show a small amount of acceptable fluctuation but are mostly in a very stable state.

Moreover, for the detector performances, the dark current was similar to in other works [38], while the photocurrent was much lower than others [39]. The low dark photocurrent might be caused by the poor substrate quality of the diamond bulk, which contained many impurities and defects, leading to the increase in the recombination of the carriers.

**Figure 6:**
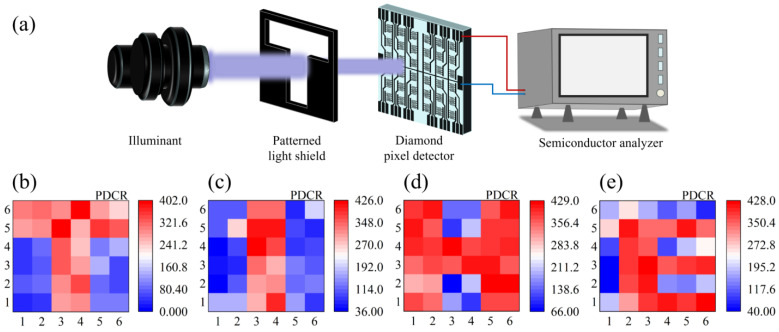
(**a**) Schematic of the imaging system employing the diamond photodetector as a sensing pixel under 50 V bias. (**b**–**e**) Images obtained from the imaging system.

The imaging performance of the all-carbon diamond pixel detector was further evaluated. The imaging system includes a light source, a polyester mask, the all-carbon diamond pixel detector, and a semiconductor analyzer, as shown in Figure 6a. The polyester visor is a black polyester mask that blocks the transmission of the 222 nm UV light, except for the hollow-letter patterns “T, I, H, and E” in the center. The polyester visor was affixed to the surface of the diamond pixel detector to generate the letter images when exposure to 222 nm UV light. The photocurrent signals of 36 pixels were read sequentially by the imaging acquisition system [40]. After eliminating the effect of the dark current of each pixel unit, the normalized photoresponse signal (*PDCR*) is obtained, as shown in Figure 6b–e. The image array exhibits high contrast, clearly delineating irradiated from unirradiated areas. The imaging boundaries are distinct, and the image aligns well with the object, suggesting that the single-crystal diamond photodetector, functioning as a sensing pixel, can produce high-fidelity images.

## 4. Conclusions

In this work, an all-carbon pixel detector was fabricated on a diamond surface with graphene as electrodes. Owing to the direct in situ sp^3^-to-sp^2^ conversion with nickel catalyst, the graphene electrodes achieved good Ohmic contact on the diamond surface with a specific contact resistance of 6.73 × 10^−5^ Ω·cm^2^. The diamond detector achieved a low dark current of 10^−11^~10^−12^ A and *PDCR* of 10^2^. The measured responsivity and detectivity of the diamond detector were about 1.35 × 10^−4^ A/W and 5.0 × 10^10^ Jones. Moreover, the fabricated 36 detector units showed good uniformity in photoresponse and successfully realized the UV imaging when exposed to UV letter images; the significant current difference between illuminated and non-illuminated pixels enables the current mapping to precisely match the pre-designed shadow mask pattern. As illustrated in Figure 6b,e, the array device exhibits a clear “T, I, H, and E” pattern, with a current contrast exceeding approximately 10^2^. This study provides a new method for fabricating diamond-based pixel detectors based on graphene/diamond heterostructures. However, the photo performance of the device needs more improvement. A high-quality diamond substrate with less defects and few impurities should be grown to improve the photocurrent and responsivity, and more advanced micro/nano-device fabrication technology can be applied to increase the pixel arrays and imaging ability.

## Data Availability

The original contributions presented in the study are included in the article, further inquiries can be directed to the corresponding authors.

## Supplementary Materials

The following supporting information can be downloaded at https://www.mdpi.com/article/10.3390/ma18061222/s1. Figure S1: Physical diamond solar-blind pixel photodetector; Figure S2: (a) Statistical graph of dark currents and photocurrents of diamond photodetector 6 × 6 array at 10 V. (b) The light/dark current ratio of the devices.
