# Supplementary material for: A High-Performance All-Carbon Diamond Pixel Solar-Blind Detector with In Situ Converted Graphene Electrodes
Source: Materials (Basel). 2025 Mar 10;18(6):1222. doi: 10.3390/ma18061222 (PMC11943947; doi:10.3390/ma18061222)
Supplement: Supplementary file 1 [file materials-18-01222-s001.zip › materials-3466508-supplementary.pdf]

Supplementary Information

A High-Performance All-Carbon Diamond Pixel Solar-Blind Detector with In Situ Converted Graphene Electrodes

Mingxin Jiang [1,2], Zhenglin Jia [2], Mengting Qiu [4], Xingqiao Chen [2], Jiayi Cai [2], Mingyang Yang [2], Yi Shen [2], Chaoping Liu [2], Kuan W. A. Chee [5], Nan Jiang [2,3], Kazuhito Nishimura [2,3], Qingning Li [1,*], Qilong Yuan [2,*] and He Li [2,3,*]

[1] School of Material Science and Engineering, Guilin University of Electronic Technology, Guilin, Guang-xi 541004, People's Republic of China; jiangmingxin@nimte.ac.cn

[2] State Key Laboratory of Advanced Marine Materials, Ningbo Institute of Materials Technology and Engi-neering, Chinese Academy of Sciences, Ningbo 315201, China; jiazhenglin@nimte.ac.cn (Z.J.); chen-xingqiao@nimte.ac.cn (X.C.); caijiayi@nimte.ac.cn (J.C.); yangmingyang@nimte.ac.cn (M.Y.); shenyi@nimte.ac.cn (Y.S.); liuchaoping@nimte.ac.cn (C.L.); jiangnan@nimte.ac.cn (N.J.); kazuhitonishimura@nimte.ac.cn (K.N.)

[3] Center of Materials Science and Optoelectronics Engineering, University of Chinese Academy of Sciences, Beijing 100049, China

[4] Southwest Institute of Technical Physics, Chengdu, 610041, China; 18183312062@163.com

[5] National Laboratory for Physical Sciences at Microscale, University of Science and Technology of China, He-fei 230026, China; Kuan.chee@cantab.net

[*] Correspondence: liqnkm@guet.edu.cn (Q.L.); yuanqilong@nimte.ac.cn (Q.Y.); lihe@nimte.ac.cn (H.L.)

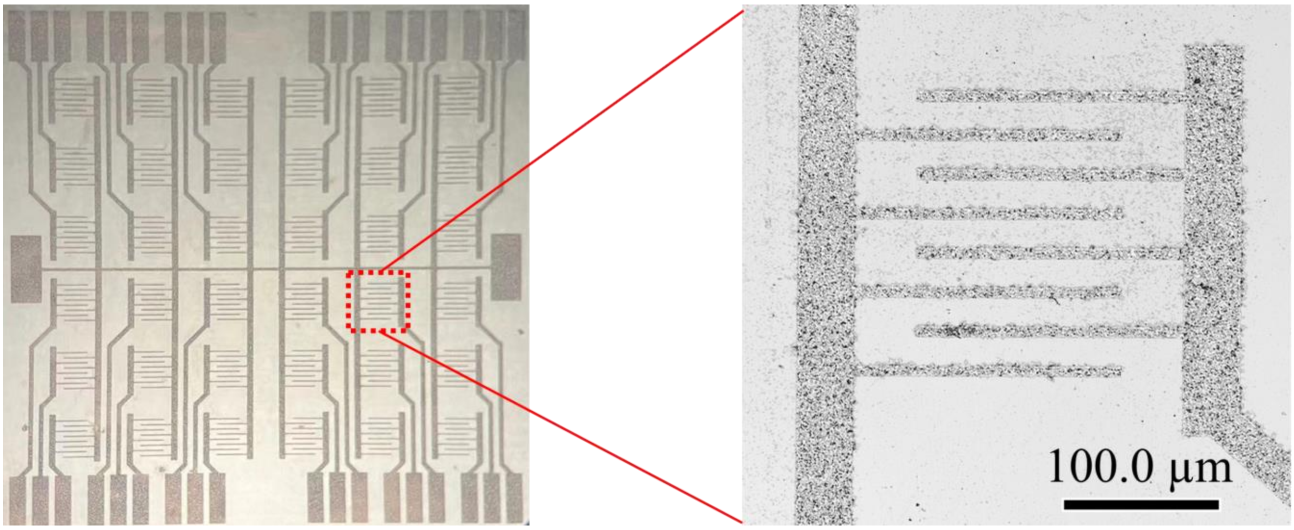

Figure S1. Physical diamond solar-blind pixel photodetector.

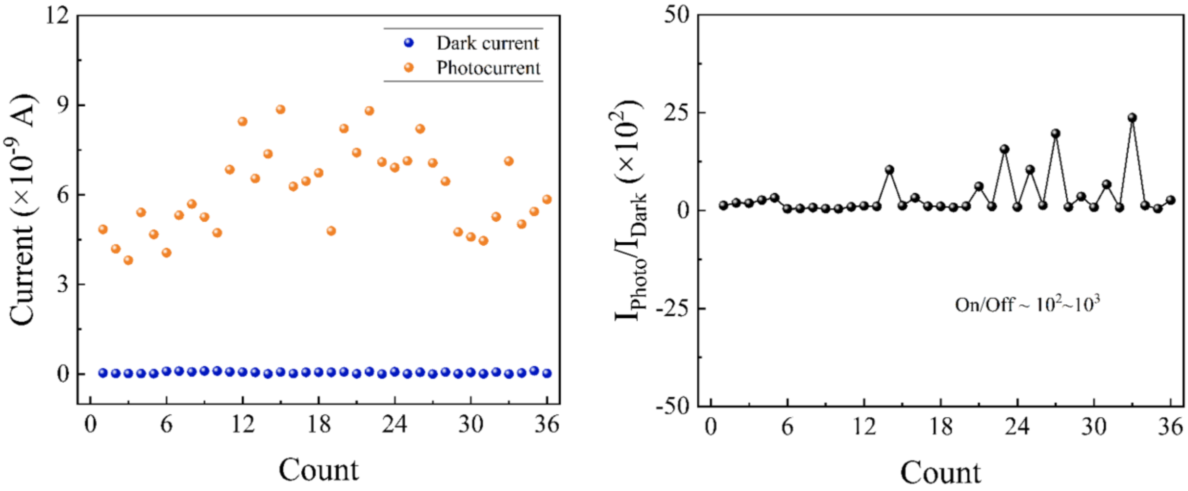

Figure S2. (a) Statistical graph of dark currents and photocurrents of diamond photodetector 6×6 array at 10 V. (b) The light/dark current ratio of the devices.
